# Performing high flexion activities does not seem to be crucial in developing early femoral component loosening after high-flexion TKA

**DOI:** 10.1186/s12891-015-0812-y

**Published:** 2015-11-16

**Authors:** Chul-Won Ha, Chandramohan Ravichandran, Choong-Hee Lee, Jun-Ho Kim, Yong-Beom Park

**Affiliations:** Department of Orthopedic Surgery, Samsung Medical Center, Sungkyunkwan University School of Medicine, 81 Irwon-ro, Gangnam-gu, Seoul 135-710 South Korea

**Keywords:** Total knee arthroplasty, High flexion activity, Aseptic loosening, Femoral component

## Abstract

**Background:**

It is still unclear whether high flexion (HF) activities correlated with the early loosening of the femoral component and whether HF activities are possible. We investigated what is the capability for performing various HF activities, and whether high flexion activities increase the chance of aseptic loosening after HF-TKA.

**Methods:**

We retrospectively analysed 260 patients who underwent HF-TKA using the NexGen LPS Flex between 2001 and 2009. The mean follow-up was 6.7 years (range, 5–13). We evaluated range of motion, Knee Society scores, WOMAC, and serial radiographs for aseptic loosening. Responses to questions on individual HF activities were recorded on 5-point Likert scales based on difficulty (0–4). Patients were divided two groups based on their responses to squatting and kneeling, which were important weight-bearing HF activities in Asian population (HF group vs. non-HF group) for comparisons of aseptic loosening and clinical outcomes.

**Results:**

More than 80 % of patients positively responded for various HF activities. The capability of HF activities showed that cross-legged sitting, squatting, and kneeling were 97.7, 51.1 and 52.7 % at the latest follow-up, respectively. Aseptic loosening was identified in two tibial components (0.8 %) but none in femoral components in non-HF group. There was no significant difference of aseptic loosening based on HF activities (0.8% vs. 0%, *p* = 0.063).

**Conclusions:**

The results of this study suggest that HF activities do not seem to be associated with aseptic loosening of femoral component after HF-TKA.

## Background

The successful pain relief and long-term durability after conventional total knee arthroplasty (TKA) [1] lead patients to expect the feasibility of more demanding activities. With a small modification in surgical technique and implant design, the high-flexion (HF) TKA was introduced to accommodate superior range of motion (ROM) compared to conventional TKA [2]. ROM after TKA is an important determinant of activity level and knee function [3]. Since HF activities are an integral part of many activities of daily living (ADL) in Asian and Middle East population, and owing to increasing patient demands for continuing their HF activities after TKA, HF-TKA is surpassing conventional TKA and is being increasingly preferred by surgeons also as they can safely accommodate knee flexion greater than 135° and can support a knee flexion even up to 155°.

There was still some controversy about clinical result and complication after HF-TKA. Some recent studies performed on Asian population has reported an alarming, increased incidence of aseptic loosening of femoral components in HF-TKA and attributed it to HF activities done by those patients after HF-TKA [4–6]. An in-vitro study has also hypothesised that HF-TKA designs have a greater risk of femoral component loosening [7]. However, in other recent studies, there was no decreased rate of survivorship after HF-TKA [8,9]. In some studies, HF-TKA showed superior ability to do squatting, kneeling, crossed legged sitting which were the three most important weight bearing HF activities in Asian population requiring a knee ROM between 111 and 165°, compared to conventional TKA [10,11]. Nevertheless, many surgeons are still concerned that some reports of early loosening associated with HF activities after HF-TKA does not exclude a higher risk of loosening in the longer term. Indeed the capability of performing individual HF activities after a HF-TKA is not detailed in any recent literature.

Therefore, we investigated what is the capability for performing various HF activities, and whether HF activities increase the chance of aseptic loosening after HF-TKA.

## Methods

We retrospective analyzed prospectively collected data of 294 patients underwent HF-TKA between January 2001 and December 2009. Among 294 patients, 34 patients were excluded (seven patents died and 27 were lost to follow up), leaving 260 patients for this study (88.4 %). Decision for HF-TKA were based on considering their preoperative ROM, life style and activity level, knee alignment, deformities, patient expectation after surgery [12]. We performed HF-TKA for patients with preoperative maximal flexion more than 100° or more. There were 212 women and 48 men with age of 69 years (range, 57–83 years) at the time of surgery. The body mass index was 27.0 kg/m^2^ (range, 20.5–34.1 kg/m^2^). Minimum follow-up was 5 years (mean, 6.7 years; range, 5–13 years). This study was performed with the approval of the institutional review board of our hospital (Samsung Medical Center 2014-01-065). All participants gave their written informed consent to assessing and using their data.

The senior author (C-WH) of this study performed all the TKAs using standardized technique as described elsewhere [13]. The cement was applied directly on the anterior flanges and distal cut surface of femur and on the posterior facet of the femoral component [14]. Posterior femoral osteophytes were carefully removed with knee in full flexion to aid in increased postoperative flexion. Patella was not resurfaced in this cohort. All surgeries in our study were done using NexGen Legacy Posterior-Stabilized Flex fixed bearing implant (Zimmer, Warsaw, IN, USA). The same postoperative rehabilitation program was used for all patients. Briefly, a closed suction drain was used for 2 days, and ankle pump exercises were commenced immediately after surgery. On the second postoperative day, a continuous passive motion machine was applied at a range of motion tolerated by the patient, and ambulation with a walker was encouraged. Patients were also encouraged to perform active and assisted knee flexion, and quadriceps setting exercises, and straight leg raising exercise against gravity. After the rehabilitation period, HF activities were allowed as tolerated. Even weight-bearing HF activities, such as, squatting and kneeling were not prohibited, if patients needed to perform these activities.

Patients were followed up at 1, 3, 6 months, 1 year after surgery and annually thereafter. Clinical outcomes were evaluated using ROM, American Knee Society knee scores (KSKS) and function scores (KSFS), and Western Ontario and McMaster Universities osteoarthritis index (WOMAC) scores. Non weight bearing passive flexion angles were measured in the supine position by independent examiner and calculated using two reference lines, a femoral line (from the lateral epicondyle of the distal femur to the tip of the greater trochanter) and a line from the tip of the fibula head to the tip of the lateral malleolus. Functional outcomes for HF activities were evaluated using a self-administered questionnaire (in accordance with 5-point Likert scales based on difficulty, 0–4 with zero being no discomfort and four being extreme difficulty), which consisted of seven HF activities that addressed whether the operated knee permitted HF related activities, such as, ascending and descending stairs, sitting or rising from a low chair, sitting or rising from the floor, cross-legged sitting, squatting, and kneeling [13]. Responses to each question were scored according to five grades of difficulty for a particular activity (Grade 0: no difficulty, grade 1: mild difficulty, grade 2: moderate difficulty, grade 3: severe difficulty and grade 4: extreme difficulty (unable to do)). Levels 0, 1, and 2 were considered positive responses and levels 3 and 4 were considered negative responses.

For identifying aseptic loosening, radiographic evaluations were done based on the American Knee Society roentgenographic evaluation and scoring system. Full length and standing anteroposterior, lateral and Merchant’s view were acquired at each follow-up visit, and assessed for limb alignment, component positioning, and for any features of loosening. Widths of radiolucent lines were measured along the seven zones on lateral radiographs of the femur, seven zones on anteroposterior radiographs of the tibia, and three zones on lateral radiographs of the tibia. Any radiolucent lines identified were compared with the previous follow-up x-rays to classify them as progressive or non-progressive lines. Aseptic loosening is defined as radiolucency greater than 2 mm width, interval increases in the width of an existing radiolucency, cement fracture, and prosthesis migration [15]. A comparison was also made between two groups (HF group and Non-HF group) divided based on their responses to squatting and kneeling (two of the most important weight bearing HF activities done in Asian population) for evaluation in loosening rates and functional scores. Both squatting and kneeling activities are known to impose high contact stress at the posterior articular surface in both normal as well as replaced knee [16], hence if at all the concept of HF activities leading to aseptic loosening is valid, the patients doing these activities will be affected first.

### Statistical analysis

Paired t-test was used to determine the difference between preoperative and postoperative values of all continuous outcome variables (ROM, KSKS, KSFS, and WOMAC). To evaluate the effect of HF activities on the aseptic loosening, we grouped patients according to feasibility of squatting and kneeling (HF group and non-HF group). A Fisher’s exact test was used for finding any statistically significant difference in radiographic loosening rates between HF group and non-HF group. Independent t-test was used for comparison between the two groups. The significance level was set at 0.05. All statistical analyses were performed with SAS 9.3 (SAS Institute, Cary, NC, USA).

## Results

The following percentages of patients responded positively (≤Grade 2) to questions regarding their abilities to perform high flexion activities: 96.9 % for ascending stairs, 96.9 % for descending stairs, 96.3 % for sitting or rising from a low chair, 80.8 % for sitting or rising from the floor, 97.7 % for cross-legged sitting, 51.1 % for squatting, and 52.7 % for kneeling (Table 1).

**Table 1:** The capability of HF activities at the latest follow-up^a^

	Ascending stairs	Descending stairs	Sitting or rising from a low chair	Sitting or rising from the floor	Cross-legged sitting	Squatting	Kneeling
Grade 0	34 (13.1)	15 (5.8)	71 (27.3)	13 (5.0)	71 (27.3)	21(8.1)	0(0)
Grade 1	169 (65.0)	119 (45.8)	43 (57.7)	90 (34.6)	98 (37.7)	55(21.2)	22(8.5)
Grade 2	98 (18.8)	118 (45.4)	150 (12.3)	107 (41.2)	85 (32.7)	57(21.9)	115(44.2)
Grade 3	8 (3.1)	8 (3.1)	7 (2.7)	50 (19.2)	6 (2.3)	105(40.4)	59(22.7)
Grade 4	0 (0)	0 (0)	0 (0)	0 (0)	0 (0)	22(8.5)	64(24.6)
Positive^b^	252 (96.9)	252 (96.9)	253 (96.3)	210 (80.8)	254 (97.7)	133 (51.1)	137 (52.7)
Negative^c^	8 (3.1)	8 (3.1)	7 (2.7)	50 (19.2)	6 (2.3)	127 (48.9)	123 (47.3)

*HF* high flexion

^a^Data are given as number (percentage). HF activities were evaluated using a self-administered questionnaire [13]. Grade means degree of difficulty for a particular activity. Grade 0: no difficulty, grade 1: mild difficulty, grade 2: moderate difficulty, grade 3: severe difficulty and grade 4: extreme difficulty (unable to do)

^b^Grade 0, 1, and 2 were considered positive responses

^c^Grade 3 and 4 are considered negative responses

No femoral component loosening was encountered. There was revision of three knees (three patients), two of them for aseptic loosening in zone one of tibial component with tibial subsidence and one for instability in non-HF group and all patients were at follow up of 5 years or above when loosening was identified. All three patients were revised with a varus-valgus constrained prosthesis and all three were negative responders for squatting and kneeling. There was no significant difference in loosening rates between the compared groups (*p* = 0.063). There was a statistically significant difference in ROM and clinical scores between HF group and non-HF group at latest follow-up (Table 2).

**Table 2:** Comparison between groups based on squatting and kneeling of HF activities^a^

	HF group (*n* = 122)	Non-HF group (*n* = 123)	*p* value
Preoperative			
Age (years)	69.6 ± 6.4	70.2 ± 5.4	0.386
BMI(kg/m^2^)	26.8 ± 2.4	27.2 ± 3.1	0.260
Maximum flexion	124.1 ± 14.7	121.5 ± 15.3	0.162
KSKS	50.1 ± 12.1	48.6 ± 11.5	0.316
KSFS	58.3 ± 12.5	56.1 ± 12.2	0.155
WOMAC	42.4 ± 8.3	43.8 ± 11.5	0.203
Postoperative			
Maximum flexion	141.9 ± 7.5	136.1 ± 8.2	<0.001
KSKS	92.4 ± 2.6	90.5 ± 3.7	<0.001
KSFS	96.2 ± 4.6	93.1 ± 4.6	<0.001
WOMAC	8.8 ± 2.7	13.9 ± 3.8	<0.001

*HF* high flexion, *BMI* body mass index, *KSKS* Knee Society knee score, *KSFS* Knee Society function score, *WOMAC* Western Ontario and McMaster Universities osteoarthritis index

^a^Data are given as mean (SD)

**Fig 1:**
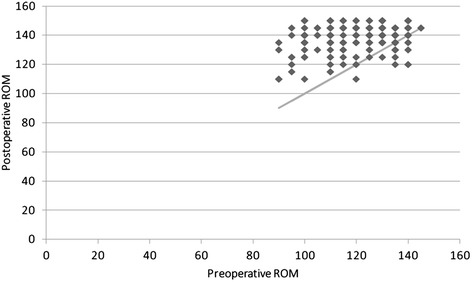
A scatter plot of change of ROM between the preoperative and postoperative state. The portion above linear line means improvement of ROM from preoperative to postoperative state. The plot reveals that postoperative ROM is overall greater than preoperative ROM

The mean flexion improved from 122.5° ± 14.9° preoperatively to 138.4° ± 11.8° at the latest follow-up (Fig. 1). Mean KSKS and KSFS scores improved from 49.1 ± 12.3 and 57.4 ± 12.3 preoperatively to 91.4 ± 3.3 and 94.5 ± 4.8 at the latest follow-up, respectively. Mean WOMAC score improved from 43.4 ± 8.8 to 11.5 ± 4.2.

## Discussion

Patient satisfaction after TKA is primarily determined by patient’s expectation about surgery and their chances of return to activities similar to the pre-arthritic stage of their life [17]. Since satisfactory pain relief after TKA is proven beyond any doubts, now-a-days many Asian patients are changing their expectations to achieve a superior ROM after TKA so as to safely perform squatting, kneeling and other HF activities that form a part of their ADL. It is for this reason that HF-TKA is gaining popularity among surgeon in Asia, Middle East. Despite these current trends, some recent in vivo and in vitro studies have questioned the long term durability of HF-TKA in those doing HF activities [4–7,18]. Those studies have attributed the increased loosening rates to the HF activities performed after HF-TKA. These alarming reports along with the facts that HF activities causes increase peak stress on articular surfaces of both replaced and nonreplaced knees (evident by increased incidence of osteoarthritis in Asian patients whose ADL involve HF activities), has led many surgeons in to a dilemma of choosing a HF design in Asian population. These study results may also lead surgeons to disallow HF-TKA patients doing HF activities overcoming the fact that the main reason for performing HF-TKA in Asian patients is for them to do HF activities after surgery. Hence we investigated what percentage of patients were capable of doing HF activities, and whether HF activities lead to increased loosening especially of femoral component and after HF-TKA.

More than 80.8 % of patients in this study positively responded for various HF activities excepting weight-bearing HF activities, indicating HF-TKA has allowed these patients to indulge in ADL that require deep knee bending. The capability of patients for performing some routinely used HF activities after HF-TKA is not reported dedicate in any previous studies regarding HF-TKA. As far as we know, our study is the first of its kind to analyse what percentage of patients were doing different HF activities and their responses for questionnaires on routinely used HF activities. Approximately 50 % of patients in this study, low percentage than other HF activities including questionnaire, responded positively to questions regarding the capability of squatting and kneeling. However, Squatting and kneeling are more high demanding activities than other HF activities since they are accompanied by weight bearing. Thus these two HF activities affected not only knee flexion but also hip and ankle motion [19]. Indeed, the kinematics of knee between weight bearing and non-weight bearing is different [20]. Therefore, we believed that HF-TKA was useful for HF activities after TKA.

Our study reveals favorable results for HF-TKA considering femoral component loosening. Some surgeons are still concerned that there might be greater risk of developing early loosening after HF-TKA especially in a longer term follow-up. Han et al. [4] reported a high incidence of early loosening of the LPS-Flex femoral component within 4 years, and noted that there were significantly more patients in the loosening group whose knees allowed squatting, kneeling, or cross-legged sitting postoperatively in the loosened group (85 %) than in the well-fixed group (49 %). They suggested these HF activities were the cause of early loosening of the femoral component. However, others studies with long-term follow-up reported good survival rates of 0–1.3 % for mechanical failure after HF-TKA [8,9]. These results indicated the implant to be a safe choice for Asian patients doing HF activities. Our results of comparison between HF group and non-HF group also revealed that in spite of the former group having better knee scores, we observed no difference in loosening rates between them. The rate of aseptic loosening of tibial component was reported as approximately 1 % after conventional TKA [21]. In the current study, there was no case of tibial component loosening in the HF group. Two cases of aseptic loosening in tibial component were observed only in the non-HF group (0.8 %). Therefore, HF activities did not seem predispose an early tibial component loosening after HF-TKA. This finding is consistent with the previous reports regarding early loosening after HF TKA [8,22]. Hence, the results of this study reinforce our hypothesis that HF activities are not a predisposing factor for early aseptic loosening after HF-TKA. We consider that the femoral implant bone cementing technique may have been responsible for the early loosening. King and Scott [23] demonstrated the importance of cementing the posterior condyles and noted that an inadequate cementing at this area resulted in premature aseptic loosening. We placed bone cement on the posterior aspect separately, which, we believe, resulted in complete filling and firm fixation of the femoral prosthesis, especially in the posterior condylar region.

Our study had some limitation. First, we did not compare the outcomes from HF-TKA and conventional TKA. But this study provides insight for the capability of HF activities after HF-TKA in Asian population which required HF activities in ADL. Second, the frequency of doing HF activities was not evaluated. A combination of the presence of HF activities and the frequency of doing HF activities would potentially contribute to the component loosening. However, the frequency of activities was difficult to measure quantitatively. Third, the load under ROM was not specifically evaluated along with the ROM itself. High stress is known to be generated by large net quadriceps moment and net posterior force during loading in high flexion angles [16]. However, the quantitative measurement of the load at specific high flexion activities in each patient is very difficult. Thus, we tried to assess the overall effect of performing high flexion activities by using the questionnaire in this study. Fourth, our study included only a specific HF design. Hence our results may be limited to generalize to other HF designs. Fifth, the majority of patients were only in a mid-term follow up; 5–6 years (73.5 %, 191/260). Nevertheless, we believed that the results of this study were meaningful based on the fact that other studies reported premature failure with HF-TKA in short-term follow-up period. Indeed we focused on the association of HF activities and femoral component loosening by group comparison. Sixth, the study cohort was nonconsecutive series. We underwent HF-TKA only to patients with 100° or more of preoperative knee flexion, because preoperative flexion was known as a predominant determinant of postoperative flexion. Seventh, the tibial slope affecting ROM was not separately evaluated in this study. Not only the femoral component geometry but also the tibial slope have significant effect on the ROM after TKA. As we tried to put the tibial component with about 3° of posterior slope in every case of this study cohort, a few degree of differences in the tibial slope does not seem to significantly affect the result of this study. Finally, ascending and descending stairs were included in HF questionnaire. Since these two activities form an important and frequently used ADL in Asian life style and produce very high compressive loads on knee joints, hence can lead to polyethylene wear and loosening [24].

## Conclusions

In this study, HF activities after HF TKA were not associated with early components loosening, and a majority of patients can perform different HF activities after HF-TKA. Some studies performed in Asia have reported high incidence of premature aseptic loosening of femoral components in HF-TKA and attributed it to high flexion activities done by those patients after HF-TKA [4–6]. In contrast, other studies have reported low incidence of aseptic loosening even minimum 5 years follow-up (Table 3). These findings suggest that HF activities do not seem to increase incidence of aseptic loosening of femoral component after HF-TKA.

**Table 3:** Studies with mid-term follow-up after HF-TKA

Study	Number of knees	Minimum follow up (years) (range)	Preoperative flexion (°)	Postoperative flexion (°)	HF activity assessment	Aseptic loosening (number [%])
Kim et al. [8]	100	10 (10 to 10.6)	125	135	No	0
Endres and Wilke [25]	79	5 (All 5)	82	122	No	0
Seng et al. [26]	36	5 (All 5)	123	128	No	0
Tarabichi et al. [27]	152	5 (All 5)	125	140	No	1 (0.5)
Tanavalee et al. [22]^a^	178	6 (6 to 7.3)	138	135	No	0
Wohlrab et al. [28]	19	5 (All 5)	106	117	No	1 (3)
Current study	260	5 (5 to 13)	123	138	Yes	2 (0.8)

*HF-TKA* high flexion total knee arthroplasty, *HF* high-flexion

^a^131 knees had adequate radiographs were included for evaluation

## Availability of data and materials

Not applicable.

## Abbreviations

ADL: activities of daily living
HF: high flexion
KSFS: Knee Society function score
KSKS: Knee Society knee score
ROM: range of motion
TKA: total knee arthroplasty
WOMAC: Western Ontario and McMaster Universities osteoarthritis index
